# Artificial Substrates Coupled with qPCR (AS-qPCR) Assay for the Detection of the Toxic Benthopelagic Dinoflagellate *Vulcanodinium rugosum*

**DOI:** 10.3390/toxins15030217

**Published:** 2023-03-11

**Authors:** Aurélien Bouquet, Christine Felix, Estelle Masseret, Coralie Reymond, Eric Abadie, Mohamed Laabir, Jean Luc Rolland

**Affiliations:** 1 MARBEC, Université de Montpellier, CNRS, IFREMER, IRD, 87 Avenue Jean Monnet, 34200 Sète, France; aurelien.bouquet@ifremer.fr (A.B.);; 2 MARBEC, Université de Montpellier, CNRS, IFREMER, IRD, Place Eugène Bataillon, 34095 Montpellier, France; 3 IFREMER, Biodivenv, 79 Route de Pointe Fort, 97231 Martinique, France

**Keywords:** artificial substrate, PCR, detection, *Vulcanodinium rugosum*, toxins, benthopelagic

## Abstract

*Vulcanodinium rugosum* is an emerging benthopelagic neuro-toxic dinoflagellate species responsible for seasonal Pinnatoxins and Portimines contaminations of shellfish and marine animals. This species is challenging to detect in the environment, as it is present in low abundance and difficult to be identified using light microscopy. In this work, we developed a method using artificial substrates coupled with qPCR (AS-qPCR) to detect *V. rugosum* in a marine environment. This sensitive, specific and easy-to-standardize alternative to current techniques does not require specialized expertise in taxonomy. After determining the limits and specificity of the qPCR, we searched for the presence of *V. rugosum* in four French Mediterranean lagoons using artificial substrates collected every two weeks for one year. The AS-qPCR method revealed its occurrences in summer 2021 in every studied lagoon and detected cells in more samples than light microscopy. As *V. rugosum* development induces shellfish contamination even at low microalga densities, the AS-qPCR method is accurate and relevant for monitoring *V. rugosum* in a marine environment.

## 1. Introduction

*Vulcanodinium rugosum* is a benthopelagic toxic dinoflagellate first identified by Nezan and Chomerat (2011) in Ingril lagoon (Southern France). It is a Peridiniale and is the only species from the genus *Vulcanodinium*. Its presence was reported in sediment, on macrophytes and in water samples in many areas around the world, among which include New Zealand (Randaunu Harbor), Japan (Okinawa), Australia (Franklin Harbour), China Sea and Cuba [1,2,3,4]. *V. rugosum* was shown to produce two fast-acting toxins (FAT), Pinnatoxins and Portimines (Prtn). These toxins can accumulate in shellfish [3,4,5] and cause death after oral or intraperitoneal administration to mice [6]. They were also shown to contaminate mugilid juveniles in high concentrations [7], which suggests that they could reach higher levels of the fish food web.

Since 2011, toxins produced by *V. rugosum* have been regularly detected during the flowering season in mussels from the French lagoon of Ingril [8,9,10,11,12] with a record concentration of toxins measured in June 2015 (up to 1244 µg of PnTX G per kg of mussel flesh). It led to farming and harvesting area closure, causing economic loss [10]. Pinnatoxins are also regularly detected in other lagoons in mussels and clams within the EMERGTOX monitoring program [12]. Because *V. rugosum* blooms could threaten human health, sanitary and environmental monitoring is essential to provide early warning of imminent blooms and toxins contamination in shellfish.

Generally, current monitoring programs preventing harmful algal blooms (HAB) occurrences are based on the identification and quantification of toxic phytoplankton species through light microscopy (LM) observation of water samples or of macrophyte samples for benthic species. The LM quantification of cells from water samples displays a limit of sensitivity for species present in low abundance in the water column, such as *V. rugosum* [8,9]. This sensitivity is limited by the volume of sampled water and the volume of the sample used for identification. As for the collection of macrophytes, it displays major standardization issues for monitoring purposes due to the spatiotemporal variations in the composition and distribution of macroalgal substrates and to the preferences of microalgae for specific macrophyte substrates [13,14].

Studies described the use of artificial substrates as an alternative for benthic dinoflagellates, such as *Prorocentrum*, *Ostreopsis*, *Gambierdiscus* or *Coolia* [14,15,16,17]. These devices may be made of various materials, such as fabric or nylon strips, test tube brushes, plastic plates or fiberglass screens [14,15,16,17]. They allow to collect a greater number of cells than water samples and offer many advantages over macrophytes. At first, cell abundances can be easily normalized to a known surface area, which allows for meaningful comparisons among sites and studies. They can be deployed in any environment, independently of the availability of natural substrates or of the phytoplankton–macroalgae preferences. Finally, samples contain fewer contaminating particles than those from natural substrates. However, the identification of *V. rugosum* collected on artificial substrates by LM requires strong taxonomic skills, as this species tends to form temporary benthic cysts on substrates, making them very difficult to discern from other species [18]. The combination with qPCR could allow to overcome this issue, as it does not require any specialized expertise in taxonomy, and it was described for many other dinoflagellate species [19,20,21,22,23,24].

In this study, we developed a highly sensitive and specific method to detect *V. rugosum* in a marine environment, using an artificial substrate coupled with qPCR (AS-qPCR). The qPCR specificity was evaluated on a wide range of phytoplankton species, some of which are regularly observed in the Mediterranean Sea. Artificial substrates were used to collect cells in four Mediterranean lagoons, and qPCR was assessed to detect *V. rugosum* cells in these samples.

## 2. Results

### 2.1. qPCR Parameters and Sensitivity

**Figure 1:**
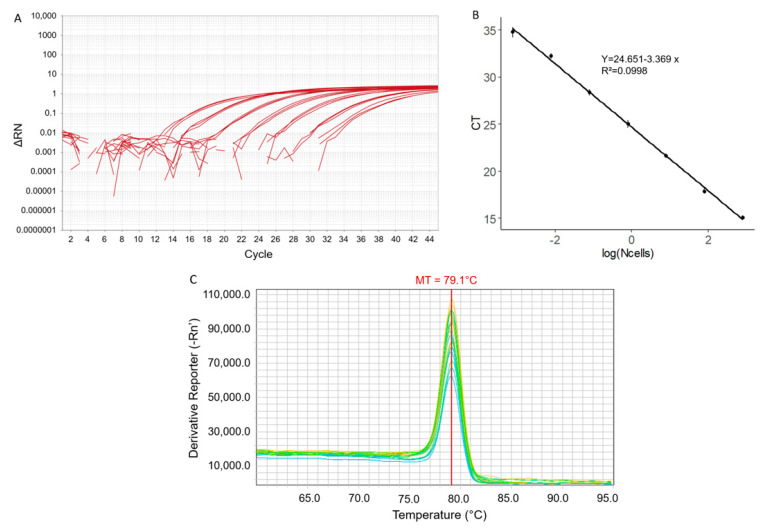
Amplification results of qPCR products from 6-fold diluted *Vulcanodinium rugosum* DNA extract: (**A**) Amplification curves plot, (**B**) standard curve, and (**C**) derivative melting curves plot. The error bars represent ± standard deviation from triplicate mean values. MT: melting temperature.

A qPCR assay was developed for the dinoflagellate *V. rugosum* using a selected designed primers pair. qPCR standard curves were established from a serial dilution of a DNA stock solution of *V. rugosum* (Figure 1A) for an optimal qPCR annealing temperature of 66 °C. A linear relationship between the Ct and the logarithm to base 10 of Equiv. cells of *V. rugosum* in 1 µL of extract was established (Figure 1B). The regression equation was y = 24.651 − 3.369x (R^2^ = 0.998), where y represents the Ct and x the log to base 10 of the numbers of equiv. cells in 1 µL of the DNA extract (Figure 1B). The efficiency of the reaction (E) was calculated to be 97.7% by using the formula E = 10(−1/m)^−1^, where m is the slope of the standard curve. From 35 to 40 cycles, the amplification results were considered positive but not quantifiable. Over 40 cycles, the results were considered negative. The limit of quantification was 0.00078 Equiv. cells corresponding to the 10^−6^ dilution. The melting temperature (MT) remained constant through the dilutions of the *V. rugosum* DNA and was calculated to be 79.1 ± 0.1 °C (Figure 1C).

### 2.2. Specificity of the qPCR

In order to determine the specificity of the PCR product, qPCR was performed on DNAs from 20 dinoflagellates species (Table 1). The use of the primers developed here showed a specific amplification with the DNAs of three *V. rugosum* strains. The amplification products size and their sequences corresponded to those expected from the large sub unit (LSU) ribosomal RNA gene of *V. rugosum* (results of sequencing not shown). No PCR amplification product was evidenced with the DNA of the other dinoflagellate species (Table 1). This result showed that the PCR developed here is specific to *V. rugosum* strains.

### 2.3. Application of AS-qPCR for Field Survey

Artificial Substrates were set up and collected every two weeks in four Mediterranean lagoons over a year period. The numbers of cells in artificial substrates samples were determined using the real-time PCR assay and LM counting method. By using LM, we observed *V. rugosum* cells in a few samples in summer 2021 in numbers ranging from 0.036 (corresponding to 1 cell observed in the counting chamber) to 0.36 cells/cm^2^ substrate in Ingril, Prévost and Vic lagoons (Table 2). No cells were observed in the Thau lagoon. By using the qPCR assay, the presence of *V. rugosum* cells was detected in the same samples as with LM, along with many other samples in summer 2021 in the four lagoons. Most of the time, *V. rugosum* was detected at numbers below the relative quantification limit (0.057 Equiv. cells/cm^2^ substrate) (Table 2). The highest relative numbers were detected during the summer of 2021 in June at Ingril and in July at Vic, (0.18 and 0.26 Equiv. cells/cm^2^ of substrate, respectively) (Table 2). The qPCR method also allowed for the detection of cells in other seasons in lagoons: in winter (February and March) in Thau and from autumn to spring (November, February and April) in Vic (Table 2).

## 3. Discussion

We developed an original methodology for the detection and quantification of the toxic dinoflagellate *Vulcanodinium rugosum* in a marine environment. This method, based on artificial substrates coupled with qPCR identification (AS-qPCR), showed great sensitivity and specificity and was shown to be relevant for the detection of this harmful species even in very low densities.

The artificial substrate characteristics and deployment modalities were adequate for the specific monitoring of *V. rugosum*. Many types of material have been tested for harmful algae monitoring, including nylon ropes, test tube brushes, plastic plates and fiberglass screens [14,15,16,17]. This study showed that large pieces of nylon plankton net allowed for the collection of *V. rugosum*. Their wide dimensions are interesting for low-density species [16], and the large 1 mm mesh reduces clogging in highly turbid environment, such as lagoons [25]. Previous studies generally described 24 to 72 h deployments of artificial substrates [14,15,16,17]. This short-term period is adequate for species that shows a diel pattern in the frequency at which they enter the water column and then resettle, and for species that easily detach from their substrate, such as *Ostreopsis* spp. or *Gambierdiscus* spp. However, it requires returning to the sampling site after a short period to retrieve samples. *V. rugosum* is known to form clusters of temporary cysts embedded in highly adherent mucous on their substrate [18], which could prolong their settlement for several days. This constitutes a main advantage for monitoring programs, as it reduces the time and expense to obtain samples. Further studies focusing on the influence of incubation time could, however, allow to optimize the cells’ accumulation on substrates.

The qPCR developed here was shown to be very specific and sensitive to *V. rugosum* strains. It allowed to detect a very low number of cells and allowed for the quantification of a broad range of cell densities. The limit of quantification (0.00078 Equiv. cells per reaction) and its efficiency (97.7%) are in the range of other studies describing the detection of dinoflagellates through qPCR [21,22]. The ability to detect less than one cell was previously attributed to the multiple copies of the ribosomal genes and was observed with many other dinoflagellates [19,24]. The sensitivity level of this molecular method is a significant benefit in the monitoring of species developing in low densities. The qPCR assay was optimized with primers specific for *V. rugosum*. They were tested on a great number of microalgae, most of them isolated from the marine environment, where *V. rugosum* blooms occur, and no cross-reaction occurred with these microalgae. In addition, many field samples showed no amplification, whereas several dinoflagellate species could be identified through LM (Figure S1). Taken together, these results show that the qPCR method allows for specific and sensitive detection of *V. rugosum* cells, even in environmental samples containing various microalgae species. The quantification results of this method depend on the number of LSRU gene copies per genome and may vary depending on the strains. Thus, the assay cannot yet be used in an absolutely quantitative manner in field samples, but it can be used to determine the relative abundance of *V. rugosum* and allows for the statistical assessment of environmental parameters associated with blooms.

The artificial substrates combined with qPCR (As-qPCR) were shown to be a powerful tool for the detection of *V. rugosum* cells in the environment. Artificial substrates are mainly described for the count of benthic species [14,15,16,17] and are thus adequate for *V. rugosum,* given its low density in water [8,9]. The use of these devices requires, however, to discriminate *V. rugosum* benthic temporary cysts, which are particularly complex to distinguish from other species with LM. This is partly due to changes in cell size and shape in response to the use of fixatives [26,27]. The high specificity of qPCR eliminated the misidentification of *V. rugosum* cells. Even if the relative numbers of cells detected were quite low, the high sensitivity allowed for detection in many more samples than LM and for the relative quantification of a minimum of 0.057 Equiv. cells/cm^2^ of substrate. Cells detected through qPCR could either be resuspended benthic cysts, vegetative cells or cells that have encysted on the substrates. This method provides thus a global idea of *V. rugosum* state in the environment. In comparison with the search of benthic species on macrophytes, it also reduces the complexity associated with the patchy distribution of cells on macroalgae, removes issues due to macroalgae distribution, and allows for an easy standardization of counting cell abundance per unit of surface area. In regard to all these considerations, AS-qPCR seems to be an efficient tool for monitoring *V. rugosum* in the environment.

The field survey by AS-qPCR highlighted the presence of *V. rugosum* mainly during summer, from May to September in the Ingril, Thau, Vic and Prévost lagoons. This result is in agreement with the temporal distribution of this species observed in the Ingril lagoon [8,9], and with the contamination of bivalves by Pinnatoxins observed during summer seasons in Ingril, Vic and Thau lagoons (EMERGTOX monitoring program) [12]. However, no studies have yet shown the presence of this species in the Prévost lagoon. The extension of the microalgae distribution area has long been studied [28,29,30,31]. Recent studies showed that *V. rugosum* cells could be translocated through ballast water, and that their temporary cysts could survive the passage through the guts of migratory grey mullets *Liza ramada* or oysters *Crassostrea gigas* [7,32,33]. These species are massively present in Mediterranean lagoons and could thus participate in *V. rugosum* cells dispersion [7,32,33,34]. Taken together, the results show the potential of *V. rugosum* to colonize Mediterranean lagoons and coastal marine ecosystems.

The colonization of lagoons could lead to new sanitary issues. Shellfish harvesting has been banned in the Ingril lagoon since 2020 because of Pinnatoxins contamination; Vic and Prévost lagoons are significant shellfish harvesting areas, and the Thau lagoon is the most important shellfish farming area of the French Mediterranean coast. *V. rugosum* was moreover detected in low quantities also in autumn and winter in the Thau and Vic lagoons. Even in low abundance, *V. rugosum* can lead to shellfish contamination that could pose human risks [10]. These results thus remind us that hazards should not be underestimated outside periods and areas currently considered to be at risk. Given the economical and potential sanitary consequences of shellfish contamination, the AS-qPCR method developed here could be a very useful tool for monitoring *V. rugosum* in the environment.

## 4. Conclusions

In this work, we developed an AS-qPCR assay to detect the benthopelagic dinoflagellate *V. rugosum* in the environment. This species shows characteristics that have made it so far very challenging to monitor in its natural habitats, as it is sparsely present in the water column and complex to be identified. The combination of artificial substrates, which could integrate both vegetative and benthic densities, and of the very sensitive and specific qPCR allowed for the identification and quantification of cells in the environment. The results highlighted observations of *V. rugosum* in several Mediterranean lagoons and showed critical and novel information regarding its distribution. Further studies should focus on the calibration of the artificial substrate incubation time. The need to standardize collection methods for benthic harmful algae is commonly recognized, but the approaches to do so are still diverse and complex. After species-specific optimizations, AS-qPCR could represent a sensitive, standardized and easy-to-implement tool applicable for many benthic or benthopelagic microalgae species monitoring.

## 5. Materials and Methods

### 5.1. Dinoflagellate Strains Culture

Phytoplankton strains (Table 1) were cultivated in Enriched Natural Sea Water (ENSW) media, composed of Thau lagoon water (kept at obscurity for several months, filtered at 0.2 μm, and autoclaved) enriched with sodium nitrate, Ferric EDTA, monosodium phosphate, vitamins and other oligo-elements [35]. Monoclonal algal strains were grown in batch mode in Greiner bio-one GmbH culture flasks at 100 μmole photon m-2.S-1 (12:12 h light:dark) at a salinity of 35 and a temperature of 22 °C to 25 °C until DNA extraction.

### 5.2. Artificial Substrates Environmental Sampling

#### 5.2.1. Sampling Area

**Figure 2:**
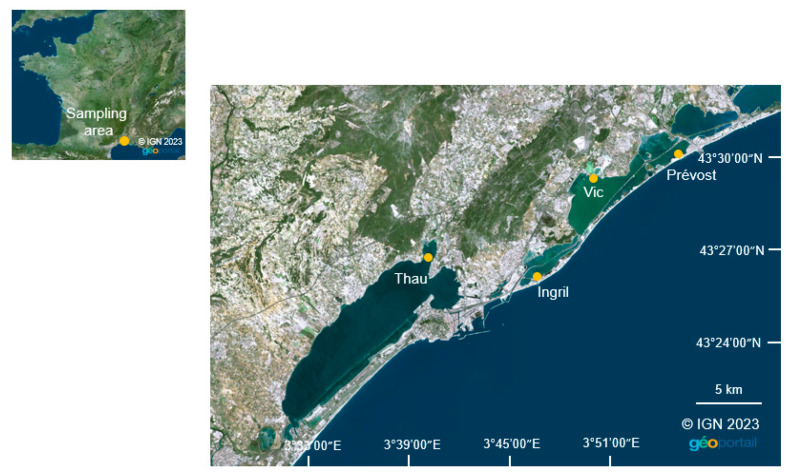
Collecting stations in Thau, Ingril, Vic and Prévost.

Collecting stations were set up in the shallow French Mediterranean lagoons of Thau (7000 ha, mean depth 4 m, station localization: 43°27′02.8″ N 3°40′01.4″ E), Ingril (685 ha, mean depth 0.6, station localization: 43°26′09.6″ N 3°46′39.8″ E), Vic (1339 ha, mean depth 1 m, station localization: 43°30′32.8″ N 3°49′20.7″ E) and Prévost (245 ha, mean depth 0.8 m, station localization: 43°30′58.7″ N 3°54′06.7″ E) [36] (Figure 2). All of these lagoons are connected through the Canal du Rhône à Sète and, apart from the Vic lagoon, they connect to the sea through inlets. The stations were all located above muddy sediments at shallow depth and near coasts, as the highest abundance of *V. rugosum* in Mediterranean lagoons was found in such environments [8].

#### 5.2.2. Deployment and Collection

One artificial substrate, made of rectangular pieces (10 cm × 14 cm) of nylon plankton net (1 mm mesh) attached to a rope with cable ties, was deposited in every station. They were collected and replaced from May 2021 to April 2022 every two weeks. The substrates were kept in plastic bottles filled with seawater. Back in the laboratory, the cells were extracted from the substrates using a modified version of the protocol developed by Jauzein et al. [15]. Briefly, the bottles were shaken vigorously for 20 s in order to detach phytoplankton cells, then the substrates were rinsed twice with 100 mL of FSW (filtered sea water). The water collected was mixed and filtered through a 125 µm filter to remove large particles, then the phytoplankton was concentrated through a 20 µm filter. Phytoplankton cells fixed on the filter were recovered using 50 mL of FSW. Then, 25 mL of water was centrifuged at 3600 rpm 30 min, and the cell pellet fixed in 1 mL of absolute ethanol and stored at −20 °C until DNA extraction. The remaining 25 mL of water was used for visual counting using inverted optical microscopy (LM) in 10 mL counting chambers after Lugol fixation, and for future *V. rugosum* cell isolation.

### 5.3. DNA Extraction

#### 5.3.1. From Phytoplankton Culture Cells

DNA was extracted from phytoplankton cultures in their exponential growth phase. The *V. rugosum* cell concentration was determined in three 1 mL subsamples fixed with Lugol′s iodine solution in which cells were counted on a Sedgwick-Rafter counting chamber under an inverted light microscope. Three 10 mL subsamples were filtered on a 10 µm polycarbonate membrane. Membranes were incubated for 12 h at 57 °C with 500 µL of buffer (Tris-Base 10 mM, NACL 100 mM, EDTA dihydrate 25 mM, SDS 0.5%, Proteinase K 0.1 mg/L). Total nucleic acids were then sequentially purified with phenol/chloroform/isoamyl alcohol (25:24:1, *v/v/v*) and absolute chloroform. DNA samples were concentrated with 5 volumes of absolute ethanol for 24 h at −20 °C. The precipitates were rinsed with 70% ethanol and resuspended in 100 µL of DNase/RNase free water. DNA quantification was performed using a spectrophotometer (NanoDrop ND-1000) to check for DNA quantity and quality (A260/A280 ratio).

#### 5.3.2. From Environmental Samples

To avoid the effect of possible interferents, DNA from environmental samples was extracted by the above phenol–chloroform method and purified on an affinity column. Thus, half the volume of each DNA extract was purified using the QIAamp^®^ DNA Mini Kit then diluted 10 times before qPCR analysis. The relative number of equivalent cells (Equiv. cells) per cm^2^ of artificial substrates was calculated as follows:

(1) $${\mathrm{Ncells}/\mathrm{cm}}^{2}\mathrm{AS}=\frac{\mathrm{Ncells}/µL\;\times \;V\;\times \;F1\;\times \;F2\;\times \;F3}{\mathrm{AS}\;\mathrm{surface}}=\frac{\mathrm{Ncells}/µL\;\times \;200\;\times \;10\;\times \;2\;\times \;2}{140}$$

where

Ncells/cm^2^AS: Equiv. cells per cm^2^ of artificial substrate (in cells/cm^2^ substrate)
Ncells/µL: Equiv. cells per µL in diluted purified DNA extracts (in cells/µL)
V: Total Volume of diluted purified DNA extracts (in µL)
F1: Factor associated with the dilution of purified DNA extracts
F2: Factor associated with the volume of DNA extract used for purification
F3: Factor associated with the volume of water used for DNA extraction
AS surface: Artificial Substrates surface (in cm^2^)

The limit of quantification in artificial substrates was calculated by applying this formula on the limit of quantification in 1 µL of *V. rugosum* DNA extract estimated with the standard curve.

### 5.4. Primers Design

**Figure 3:**
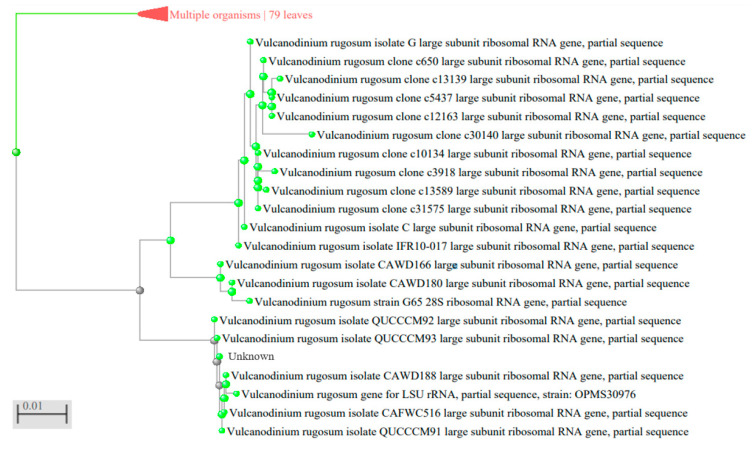
Phylogenetic distance tree of *Vulcanodinium rugosum* LSRU genes used to design PCR primers.

**Figure 4:**
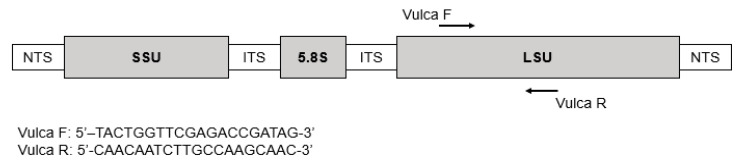
Structure of *Vulcanodinium rugosum* species rDNA and location of primers for the *V. rugosum* genus-specific PCR. NTS: non-transcribed spacer; SSU: small subunit; LSU: large subunit.

V. rugosum Large Sub Unit (LSU) ribosomal RNA genes sequences were obtained from GenBank using the Basic Local Alignment Search Tool from NCBI (National Centre for Biotechnology Information) (Figure 3, Table 3) and aligned using Multalin [37]. Specific primers were designed in a conserved region to amplify a 132 bp fragment (Figure 4). The two primers were as follows: (VulcaF; 5′ TACTGGTTCGAGACCGATAG 3′) sens and (VulcaR: 5′ CAACAATCTTGCCAAGCAAC 3′) antisens.

### 5.5. qPCR Assay

Quantitative PCR was performed in 96-well plates using a QuantStudio3 thermocycler (Applied Biosystems TM). Each well contained 5 µL CYBERgreen mix (SYBERgreen, dNTP, Polymerase, buffer), 1 µL each of the sens and antisens primers (3.33 µM), 2 µL of DNase/RNase free water and 1 µL of the DNA template. The quantification cycling protocol was as follows: an initial denaturation step at 95 °C for 5 min, followed by 45 cycles of 10 s at 95 °C, 50 to 70 °C for 10 s and 72 °C for 10 s. The melting curve profile was generated by increasing temperature from 55 °C to 95 °C at 0.5 °C /s. Amplification products were analyzed using QuantStudio3 software V1.5.2 (Applied biosystemsTM).

### 5.6. Standard Curve and Specificity

To prepare DNA templates for the construction of the qPCR standard curve, DNA extracted from a culture of V. rugosum isolated in Ingril lagoon in 2010 by Nézan [12] was serially diluted from 10^-1^ to 10^-6^ in miliQ water. The qPCR was performed on the dilutions in triplicate. Cells were considered non-quantifiable when CT was ranged between 35 and 40 and non-detectable when CT was over 40.

In order to determine the specificity of the PCR product, DNA samples from 20 dinoflagellates species were used as templates for qPCR reaction (Table 1).

### 5.7. Data Analysis

Graphics and statistical analysis were performed using R software [38].

## Figures and Tables

**Table 1:** PCR amplification results on different dinoflagellate species. Nd: not detectable.

Species	Strains and/or Origins	Collections	TM (°C)
*Fukyuoa paulensis*	RCC-6550	Roscoff Culture Collection (RCC)	Nd
*Fukyuoa* sp.	RCC-6548	RCC	Nd
*Gambierdiscus belizeanus*	RCC-6344	RCC	Nd
*Gambierdiscus carolinianus*	RCC-6338	RCC	Nd
*Gambierdiscus* spp.	RCC-6328	RCC	Nd
*Alexandrium minutum*	Britany, France	MARBEC, University of Montpellier Collection (UMC)	Nd
*Alexandrium pseudogonyaulax*	Bizerte lagoon, Tunisia	Marbec, UMC	Nd
*Alexandrium pacificum*	ACT03, Thau, France	Marbec, UMC	Nd
*Alexandrium pacificum*	Bizerte lagoon, Tunisia	Marbec, UMC	Nd
*Amphidinum carterae*	SAMS, Oban, UK	Marbec, UMC	Nd
*Coolia monotis*	CMBZT14, Bizerte, Tunisia	Marbec, UMC	Nd
*Gymnodinium catenatum*	M'diq Bay, Morocco	Marbec, UMC	Nd
*Gyrodinium impudicum*	Gulf of Tunis, Tunisia	Marbec, UMC	Nd
*Karenia selliformis*	Gulf of Gabes, Tunisia	Marbec, UMC	Nd
*Prorocentrum lima*	PLBZT14, Bizerte lagoon, Tunisia	Marbec, UMC	Nd
*Prorocentrum micans*	H4-3, Thau lagoon, France	Marbec, UMC	Nd
*Ostreopsis siamensis.*	P550, Gbraltar strait	Marbec, UMC	Nd
*Scrippsiella trochoidea*	ST17, Mellah, Algeria	Marbec, UMC	Nd
*Scrippsiella accuminata*	China Sea	Marbec, UMC	Nd
*Vulcanodinium rugosum*	IFR-VRU-01, Ingril, France	Marbec, UMC	79.1
*Vulcanodinium rugosum*	15-Ing-5.48, Ingril, France	Marbec, UMC	79.1
*Vulcanodinium rugosum*	21-Ing-1.96, Ingril, France	Marbec, UMC	79.1
*Vulcanodinium rugosum*	21-Vic-2.96, Vic, France	Marbec, UMC	79.1

**Table 2:** Detection and quantification of *Vulcanodinium rugosum* cells during one year of monitoring in four French Mediterranean lagoons: by light microscopy (LM, in cells/cm^2^ substrate) and by qPCR (relative quantification in Equiv. cells/cm^2^ substrate). Nd: not detectable.

Collection Date			Lagoons							
			Thau		Ingril		Vic		Prévost	
Year	Month	Day	LM	qPCR	LM	qPCR	LM	qPCR	LM	qPCR
2021	May	4th	Nd	Nd	Nd	Nd	Nd	Nd	Nd	Nd
		20th	Nd	Nd	0.11	<0.057	Nd	Nd	Nd	Nd
	Jun.	3rd	Nd	Nd	Nd	Nd	Nd	<0.057	Nd	<0.057
		16th	Nd	Nd	0.36	0.26	Nd	<0.057	Nd	Nd
		29th	Nd	Nd	Nd	<0.057	Nd	<0.057	0.11	<0.057
	Jul.	7th	Nd	Nd	Nd	<0.057	0.25	0.18	Nd	Nd
		27th	Nd	Nd	Nd	<0.057	0.071	<0.057	Nd	Nd
	Aug.	10th	Nd	<0.057	0.036	<0.057	0.036	<0.057	Nd	<0.057
		25th	Nd	Nd	Nd	Nd	0.036	<0.057	Nd	Nd
	Sep.	8th	Nd	Nd	Nd	Nd	Nd	Nd	Nd	<0.057
		21th	Nd	<0.057	Nd	Nd	Nd	Nd	Nd	ND
	Oct.	4th	Nd	Nd	Nd	Nd	Nd	Nd	Nd	Nd
		19th	Nd	Nd	Nd	Nd	Nd	Nd	Nd	Nd
	Nov.	3rd	Nd	Nd	Nd	Nd	Nd	<0.057	Nd	Nd
		19th	Nd	Nd	Nd	Nd	Nd	Nd	Nd	Nd
	Dec.	2nd	Nd	Nd	Nd	Nd	Nd	Nd	Nd	Nd
		16th	Nd	Nd	Nd	Nd	Nd	Nd	Nd	Nd
2022	Jan.	4th	Nd	Nd	Nd	Nd	Nd	Nd	Nd	Nd
		20th	Nd	Nd	Nd	Nd	Nd	Nd	Nd	Nd
	Feb.	3rd	Nd	Nd	Nd	Nd	Nd	<0.057	Nd	Nd
		15th	Nd	<0.057	Nd	Nd	Nd	Nd	Nd	Nd
	Mar.	1st	Nd	<0.057	Nd	Nd	Nd	Nd	Nd	Nd
		17th	Nd	Nd	Nd	Nd	Nd	Nd	Nd	Nd
		29th	Nd	Nd	Nd	Nd	Nd	Nd	Nd	Nd
	Apr.	12th	Nd	Nd	Nd	Nd	Nd	<0.057	Nd	Nd

**Table 3:** Sequences and lengths of *Vulcanodinium rugosum* LSRU genes obtained from GenBank with Basic Local Alignment Search Tool from NCBI (National Centre for Biotechnology Information).

LSRU Genes	Sequences	Sequences Lengths
Isolate G	MG826107.1	1351
Clone c650	MK236574.1	620
Clone c13139	MK236578.1	620
Clone c5437	MK236579.1	619
Clone c12163	MK236577.1	622
Clone c30140	MK236582.1	619
Clone c10134	MK236580.1	617
Clone c3918	MK236576.1	619
Clone c13589	MK236581.1	620
Clone c31575	MK236575.1	618
Isolate C	MG826106.1	928
Isolate IFR10-017	HQ622103.1	1384
Isolate CAWD166	JF267773.1	805
Isolate CAWD180	JF683380.1	788
Strain G65	JX457352.1	596
Isolate QQCCCM92	KX853181.1	856
Isolate QQCCCM93	KX853179.1	846
Isolate CAWD188	JF683382.1	788
Strain OPMS30976	LC228963.1	599
Isolate CAFWC516	KM252944.1	845
Isolate QUCCCM91	KX853180.1	847

## Supplementary Materials

The following supporting information can be downloaded at: https://www.mdpi.com/article/10.3390/toxins15030217/s1, Figure S1: Derivatives melting curve plot for A. *Vulcanodinium rugosum* DNA extract 2-fold diluted diluted, B. Field sample, Vic lagoon, 7 July 2021, C. Field sample, Vic lagoon, 2nd December 2021.
