# Supplementary material for: Artificial Substrates Coupled with qPCR (AS-qPCR) Assay for the Detection of the Toxic Benthopelagic Dinoflagellate Vulcanodinium rugosum
Source: Toxins (Basel). 2023 Mar 11;15(3):217. doi: 10.3390/toxins15030217 (PMC10055951; doi:10.3390/toxins15030217)
Supplement: Supplementary file 1 [file toxins-15-00217-s001.zip › toxins-2252011-supplementary.pdf]

Supplementary Materials: Artificial Substrates Coupled with qPCR (AS-qPCR) Assay for the Detection of the Toxic Benthopelagic Dinoflagellate *Vulcanodinium rugosum*

Aurélien Bouquet, Christine Felix, Estelle Masseret, Coralie Reymond, Eric Abadie, Mohamed Laabir and Jean Luc Rolland

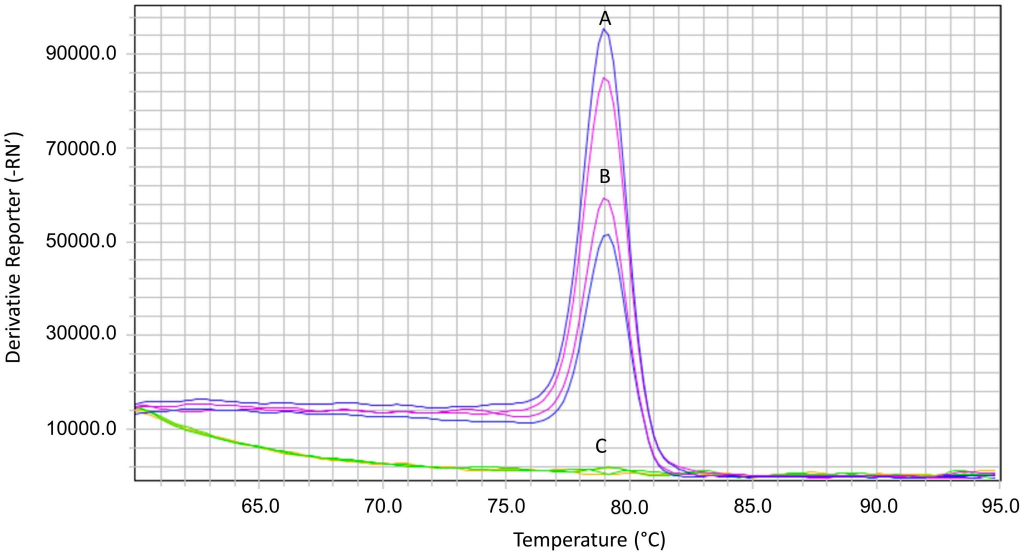

Figure S1: Derivatives melting curve plot for A. *Vulcanodinium rugosum* DNA extract 2-fold diluted diluted, B. Field sample, Vic lagoon, 7th July 2021, C. Field sample, Vic lagoon, 2nd December 2021.
